# Oxidative Stress Is Involved in the Pathogenesis of Keshan Disease (an Endemic Dilated Cardiomyopathy) in China

**DOI:** 10.1155/2013/474203

**Published:** 2013-08-26

**Authors:** Junrui Pei, Wenqi Fu, Liu Yang, Zhiyi Zhang, Yang Liu

**Affiliations:** ^1^Key Lab of Etiologic Epidemiology of Ministry of Health (23618104), Center for Endemic Disease Control, Chinese Center for Disease Control and Prevention, Harbin Medical University, Harbin, Heilongjiang Province 150081, China; ^2^School of Public Health, Harbin Medical University, Harbin, Heilongjiang Province 150081, China; ^3^School of Public Health, Shandong University, Jinan, Shandong Province 250012, China

## Abstract

Oxidative stress and selenoprotein deficiency are thought to be associated with the pathogenesis of Keshan disease (KD). However, to our knowledge, the level of oxidative stress and expression of selenoproteins have not been investigated in the myocardium of patients with KD. In this study, 8-hydroxy-2-deoxy guanosine (8-OH-dG), a marker of oxidative stress, was used to assess the level of oxidative stress, and thioredoxin reductase 1 (TrxR1) and glutathione peroxidase 1 (GPx1) were assessed to reflect the level of selenoproteins. Myocardial samples from 8 patients with KD and 9 non-KD patients (controls) were immunohistochemically stained for 8-OH-dG, TrxR1, and GPx1. The staining intensities were subsequently quantified using Olympus Image-Pro Plus 6.0 software. The data showed that the positive rate of 8-OH-dG expression in myocardial nuclei was higher in the KD group (68.6%) than that in the control group (2.4%). In addition, a positive correlation between the positive rate of 8-OH-dG and the degree of myocardial damage was observed in the KD group. The distribution of TrxR1 and GPx-1 was not associated with the distribution of myocardial damage. The expression of these two selenoproteins was higher in the control group than that in the KD group. Our study represents the first report on the expression profiles of oxidative stress and selenoproteins in the myocardium of patients with KD. The level of oxidative stress significantly increased and was positively correlated with the degree of myocardial damage in patients with KD. The selenoproteins, TrxR1 and GPx1, may have a role in the pathogenesis of KD.

## 1. Introduction

Keshan disease (KD) is an endemic cardiomyopathy of unknown cause and is named after Keshan County in Heilongjiang Province where the disease was first identified in 1935. It is clinically categorized into four groups: acute, subacute, chronic, and latent. The epidemiological characteristics of KD show a regional distribution. It is distributed in a narrow low-selenium belt from Northeast to Southwest China [1].

 There is growing evidence showing that selenium deficiency is closely related to KD. The selenium levels in soil and food in the external environment of the endemic area are significantly lower than those in the nonendemic area. Blood and hair selenium levels in patients with KD are also significantly lower than those in healthy individuals [2]. In particular, the incidence of the acute and subacute types of KD significantly decreased after selenium supplementation [2]. Researchers have proposed that oxidative stress induced by selenium deficiency plays a pivotal role in the pathogenesis of KD [3]. As a vital antioxidant element, selenium is incorporated into selenoproteins to fulfill its biological functions. Glutathione peroxidase1 (GPx1) and thioredoxin reductase 1 (TrxR1), two selenoproteins, are important members of the body's antioxidant system. They catalyze the reduction of hydrogen peroxide to eliminate harmful reactive oxygen species from the tissues and protect biological membranes and large molecular structures from oxidative damage [4]. 8-OH-dG is a modified product of oxidative DNA damage and is usually used as a biomarker for assessing oxidative stress [5].

 Previous research showed that the blood GPx activity in patients with KD was lower than that in subjects in the nonendemic area [6]. However, there has been no report on the expression of selenoproteins and the level of oxidative stress in the myocardium of patients with KD. In this study, the level of oxidative stress was evaluated by determining 8-OH-dG and the level of selenoproteins was assessed by the detection of TrxR1 and GPx1 in the myocardium of subjects with or without KD. We found that oxidative stress was detected in the myocardium of subjects with KD. A positive correlation between the positive rate of 8-OH-dG expression and the degree of myocardial damage was observed. The expression of TrxR1 and GPx1 decreased significantly in the KD group compared with the controls. Oxidative stress may be induced by a decrease in the expression of TrxR1 and GPx1 in patients with KD.

## 2. Methods 

### 2.1. Ethic Statement

The experimental protocols and procedures used in this study were approved by the Medical Ethics Committee of Harbin Medical University.

### 2.2. Tissue Samples

Tissue samples from 17 autopsies were obtained from Harbin Medical University. These tissue samples were divided into the KD group (*n* = 8) and the non-KD group (the control group) (*n* = 9) according to the pathological diagnostic criteria of KD (WS210-2001). The KD group included 4 acute KD and 4 chronic KD patients (4 males and 4 females, mean age 28 ± 6). The specimens in the control group were obtained from a non-KD area (4 males and 5 females, mean age 30 ± 5). The subjects in the control group all died in the traffic accident. All specimens were embedded in paraffin and 5 *μ*m sections were cut.

### 2.3. Antibodies

Mouse anti-human 8-OH-dG antibody (sc-66036), mouse anti-human GPx1 antibody (sc-74498), and mouse anti-human TrxR1 antibody (sc-28321) were bought from Santa Cruz Biotechnology, Inc., USA. Mouse anti-enterovirus VP1 (E3310-01) was bought from USBio Company, USA. The horseradish peroxidase-conjugated goat anti-mouse IgG and diaminobenzidine dye kit were bought from Zhong Shan-Golden Bridge Biological Technology Co., Ltd., Beijing, China.

### 2.4. Immunohistochemical Staining

Immunohistochemical staining for 8-OH-dG, TrxR1, and GPx1 was performed using standard techniques as previously described [7]. Formalin-fixed, paraffin-embedded tissue sections of 5 *μ*m thickness were deparaffinized and hydrated. Endogenous peroxidase was inactivated by covering the tissue with 3% hydrogen peroxide for 10 min. The slides were washed three times with phosphate buffer (pH 7.2; 3 min each). Heat-induced epitope retrieval was performed twice using citrate buffer (pH 6.0) and a microwave at 100°C (10 min each). After microwave irradiation, the sections were allowed to cool down to room temperature. Subsequently, the slides were washed with phosphate buffer (pH 7.2) 3 times (3 min each). The slides were blocked with 5% bull serum albumin solution at room temperature for 20 min. Tissue sections were then incubated overnight with anti-8-OH-dG antibody (1 : 100), anti-TrxR1 antibody (1 : 100), or anti-GPx1 antibody (1 : 100) in a humidified chamber at 4°C. After three washes with phosphate buffer, tissue sections were incubated with horseradish peroxidase-conjugated goat anti-mouse IgG (Zhong Shan-Golden Bridge) at 37°C for 20 min. The slides were then washed five times with phosphate buffer (3 min each). Freshly prepared diaminobenzidine substrate was added to the sections at room temperature for 3 min. The sections were then rinsed with water and counterstained with hematoxylin. Samples were dehydrated using a general protocol and sealed with neutral balsam. Immunohistochemical staining was examined by two pathologists blinded to the array composition.

### 2.5. Quantitative Analysis of the Immunohistochemistry

Observations were performed using a light microscope (BX51, Olympus, Japan) with a 40x objective (UplanSApo, Olympus). The images were captured using a digital camera (DP72, Olympus). Five photographs of each specimen were randomly taken under the same conditions including light source, color saturation, brightness, gain, and contrast. The photographs were then quantified using Image-Pro Plus 6.0 (IPP 6.0) software. Quantitative analysis of the immunohistochemistry findings was performed after color segmentation based on the fixed threshold value of hue, saturation, and intensity (HSI). The positive rate of 8-OH-dG was obtained through the number of cardiac myocytes in the view divided by the number of 8-OH-dG positive cardiac myocytes which reflected the level of oxidative stress. The quantity of TrxR1 and GPx1 was expressed as integrated optical density (IOD, area × average optical density).

### 2.6. Statistical Analysis

The data were expressed as mean ± SD. SPSS 11.0 software was used for all statistical analyses. Comparisons between two groups were evaluated using the *t*-test. *P* < 0.05 was considered statistically significant.

## 3. Results

### 3.1. The Expression of 8-OH-dG

**Figure 1:**
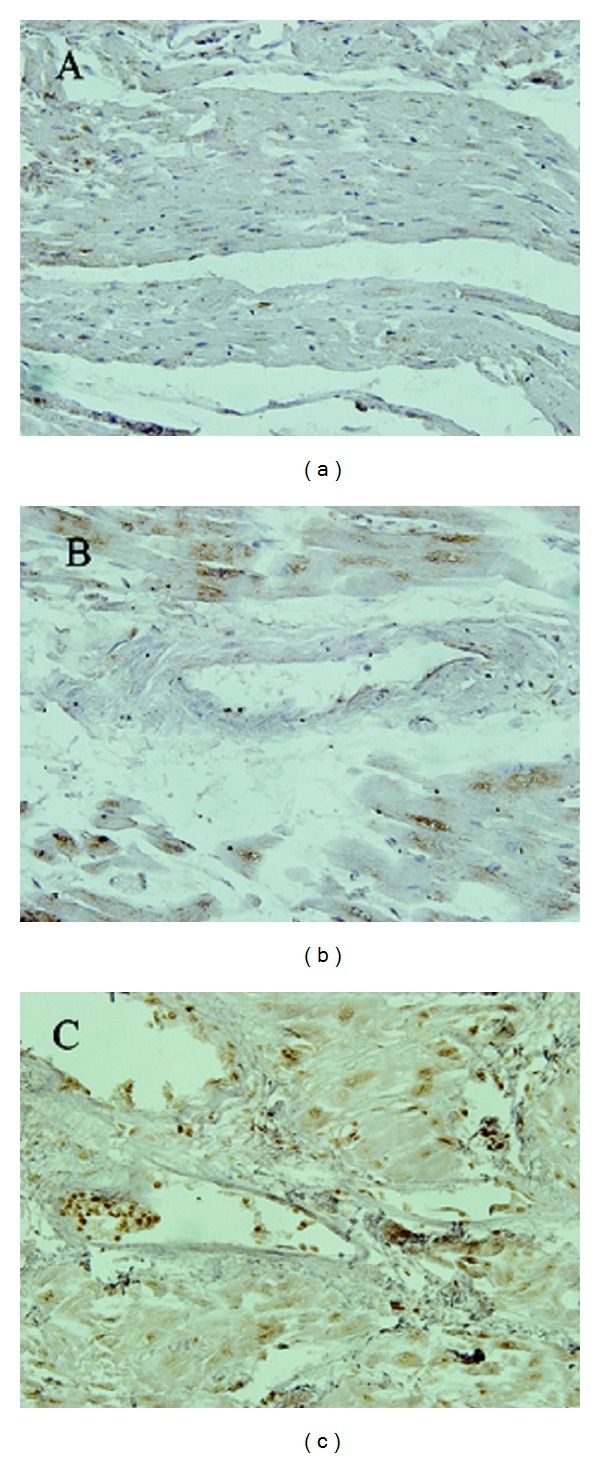
The expression of 8-OH-dG in myocardium. The positive signal of 8-OH-dG was claybank or brown and predominantly expressed in cell nuclei. The negative nuclei of 8-OH-dG were blue dyed by hematoxylin. (a) Most of myocardial nuclei are negative in the control group. (b) More than half of myocardial nuclei were positive in chronic KD. (c) Myocardial nuclei almost were positive in acute KD. 400x.

In order to detect the level of oxidative stress in the myocardium of KD patients, the expression of 8-OH-dG was determined by immunohistochemistry.  Figure 1 shows that a positive signal for 8-OH-dG was claybank or brown and was predominantly expressed in cell nuclei. In the KD group, positive nuclei were diffusely distributed in the myocardium and were strongly positive. A few nuclei were weakly positive or positive in focal myocardial necrosis. Fibrocytes were negative for 8-OH-dG. However, in the control group, myocardial nuclei were negative or weakly positive.

The data in Table 1 show that the positive rate of 8-OH-dG expression in myocardial nuclei was higher in the KD group than in the control group. In addition, the positive rate of 8-OH-dG expression in the acute KD (91.7%) was significantly higher than that in the chronic KD (53.2%). These results suggest that the level of myocardial oxidative damage was higher in the KD group than that in the control group.

### 3.2. The Expression of TrxR1 and GPx-1

**Figure 2:**
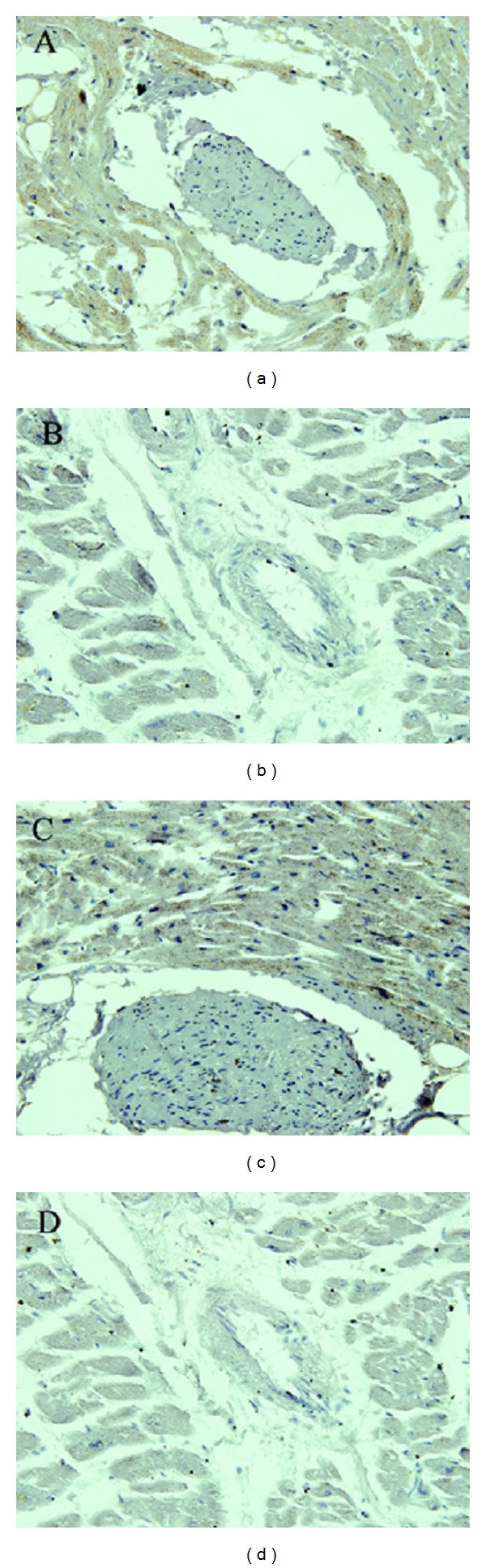
The expression of TrxR1 and GPx1 in myocardium. The positive signal of TrxR1 and GPx1 was claybank or brown and predominantly expressed in the cytoplasm. (a) Control group (TrxR1). (b) KD group (TrxR1). (c) Control group (GPx-1). (d) KD group (GPx-1). 400x.

Immunohistochemistry was used to investigate the expression of TrxR1 and GPx1 in the myocardium.  Figure 2 shows that a positive signal for TrxR1 and GPx1 was claybank or brown and predominantly expressed in the cytoplasm. The distribution of TrxR1 and GPx1 was not associated with the distribution of myocardial damage.  Table 2 shows that the IOD of these two antioxidant enzymes was higher in the control group than that in the KD group. These results indicate that a deficiency of selenoproteins existed in the myocardium of patients with KD.

## 4. Discussion

The role of oxidative stress in the pathogenesis of cardiomyopathy has been a topic of research. Studies have shown that oxidative stress may result in cardiomyopathy through activation of the apoptosis program in myocardial cells [8]. Many researchers have suggested that a deficiency in selenium is one of the principal factors responsible for KD [1]. The level of oxidative stress induced by selenium deficiency is higher in patients with KD than that in healthy subjects in non-KD areas [9]. This was corrected after selenium supplementation, and the incidence of acute KD significantly decreased [2, 10]. These results suggested that oxidative stress has a role in the pathogenesis of KD. Currently, little is known about the level of oxidative stress in the myocardium of patients with KD. 8-OH-dG is an oxidative stress biomarker and has been widely used to assess the level of oxidative injury [11]. In this study, we assessed the level of oxidative stress in the myocardium of patients with KD by detecting the expression of 8-OH-dG. It was found that the positive rate of 8-OH-dG expression in myocardial nuclei was higher in the KD group than that in the control group. In addition, a positive correlation between the positive rate of 8-OH-dG and the degree of myocardial damage was observed in the KD group. Our results provide evidence that oxidative stress is involved in the pathogenesis of KD.

TrxR1 is an isozyme of thioredoxin reductase and is expressed in the cytoplasm. It participates in the oxidative stress reaction due to its catalytic reduction activity. Research has shown that TrxR1 knockout mice showed diminished TrxR1 activity, and oxidative stress increased leading to the emergence of heart diseases [12]. To date, there are few studies on the relationship between TrxR1 and KD. The expression of TrxR1 in the myocardium of KD patients was first observed by us. It was found that the expression of TrxR1 was significantly lower in the KD group than that in the control group. This indicated that there was not only a deficiency in selenium but also a decrease in TrxR1 in the myocardium of KD patients.

GPx1, an important antioxidant enzyme, is a member of the glutathione peroxidase family and is found in the cytoplasm [13]. It was found that selenium supplementation could protect the myocardium against oxidative stress damage by increasing the activity of GPx1; therefore, the risk of cardiovascular disease decreased [14]. This research showed that GPx1 activity in peripheral blood was lower in KD patients than in healthy individuals [15]; however, the activity of GPx1 in the myocardium of KD patients is still unknown. Our results showed that GPx1 was similar to TrxR1, and the expression of GPx1 was lower in the KD group than that in the control group. The above results indicate that selenium deficiency in the myocardium of patients with KD leads to the combined deficiency of selenoenzymes such as GPx1 and TrxR1. This may decrease the capacity of the antioxidative system in the myocardium and lead to oxidative damage. This would subsequently result in the emergence of cardiomyopathy.

In summary, although the sample size was small in this study, our results strongly demonstrated that damage due to oxidative stress was present in the myocardium of patients with KD. Furthermore, a positive correlation was found between the expression of 8-OH-dG and the degree of KD myocardial damage. The selenoproteins, TrxR1 and GPx1, may be involved in the pathogenesis of KD. These results provide evidence that selenium supplementation may prevent cardiomyopathy through the oxidative stress pathway.

## Figures and Tables

**Table 1:** The expression of 8-OH-dG in subjects with or without KD.

Group	$\bar{X}\pm \text{SD}$ (%)
KD group (*n* = 8)	68.6 ± 20.4*
Control group (*n* = 9)	2.4 ± 1.5

*P < 0.05 compared with the control group.

**Table 2:** The expression of TrxR1 and GPx1 in subjects with or without KD.

Protein	Group (KD = 8, control = 9)	Mean ± SD (IOD)	*t*	*P*
TrxR1	KD group	401340 ± 59865	−28.493	*P* < 0.01
	Control group	2790300 ± 379298		
GPx 1	KD group	497590 ± 197082	−6.016	*P* < 0.01
	Control group	1348400 ± 615840		
